# A new topological descriptor for water network structure

**DOI:** 10.1186/s13321-019-0369-0

**Published:** 2019-07-10

**Authors:** Lee Steinberg, John Russo, Jeremy Frey

**Affiliations:** 1 School of Chemistry, University of Southampton, Southampton, SO17 1BJ UK; 2 School of Mathematics, University of Bristol, Bristol, UK

**Keywords:** Persistent homology, Water networks, Topological data analysis

## Abstract

Bulk water molecular dynamics simulations based on a series of atomistic water potentials (TIP3P, TIP4P/Ew, SPC/E and OPC) are compared using new techniques from the field of topological data analysis. The topological invariants (the different degrees of homology) derived from each simulation frame are used to create a series of persistence diagrams from the atomic positions. These are averaged over the simulation time using the persistence image formalism, before being normalised by their total magnitude (the L1 norm) to ensure a size independent descriptor (L1NPI). We demonstrate that the L1NPI formalism is suitable for the analysis of systems where the number of molecules varies by at least a factor of 10. Using standard machine learning techniques, a basic linear SVM, it is shown that differences in water models are able to be isolated to different degrees of homology. In particular, whereas first degree homology is able to distinguish between all atomistic potentials studied, OPC is the only potential that differs in its second degree homology. The L1 normalised persistence images are then used in the comparison of a series of Stillinger–Weber potential simulations to the atomistic potentials and the effects of changing the strength of three-body interactions on the structures is easily evident in L1NPI space, with a reduction in variance of structures as interaction strength increases being the most obvious result. Furthermore, there is a clear tracking in L1NPI space of the *λ* parameter. The L1NPI formalism presents a useful new technique for the analysis of water and other materials. It is approximately size-independent, and has been shown to contain information as to real structures in the system. We finally present a perspective on the use of L1NPIs and other persistent homology techniques as a descriptor for water solubility.

**Electronic supplementary material:**

The online version of this article (10.1186/s13321-019-0369-0) contains supplementary material, which is available to authorized users.

## Introduction

### The water network problem

Understanding the structure and dynamics of water networks is an important task in a wide variety of fields. This is due to the anomalous behaviour of water, such as the well-known density maximum. Further, these anomalies have been shown to play important roles in physical, chemical, and biological processes [1, 2]. There have therefore been many studies of simulated water systems, often looking at radial distribution functions [3] or spatial distribution functions [4]. In particular, the tetrahedral nature of local water has been investigated [5, 6].

This has led to a plethora of computational techniques for understanding water network structure. In general, these can be split into categories such as coordination number studies [3, 7–9] and graph-theoretical studies [10–13]. Both of these categories have drawbacks, namely the difficulty in interpreting data beyond nearest neighbours, and the requirement for a connectivity heuristic respectively.

Mathematical techniques drawn from topology look highly suitable to make progress in the analysis of connectivity. In particular, persistent homology is a recent development in mathematics, in the field of topological data analysis, and creates a multiscale representation of an arbitrary point cloud [14]. This is achieved by converting this point cloud into a filtration of topological structures, and observing how topological invariants change in this filtration. Persistence has found many uses in chemistry, mainly in proteins [15–22], but also as a small molecule descriptor [23, 24] or a descriptor for the analysis of crystal structures and other materials [25–30]. Furthermore, persistent homology has recently been applied to understanding water networks [31] however these methods did not take into account the dynamic nature of such systems.

In this work, we develop the ideas discussed in [31] and by the use of persistence images [32] are able to develop what we term *l*_1_-normalised persistence images (L1NPIs) which take into account the dynamic nature of the molecular dynamics simulations. These descriptors are size-agnostic, meaning they can be used between systems with vastly different numbers of water molecules, and are well-suited for machine learning techniques. We apply this technique to a range of atomistic water models and a coarse-grained Stillinger–Weber (SW) potential [33], and using this technique are able to not only distinguish between these models, but relate these differences to the underlying water network. We lastly present a perspective as to how this technique can be used to understand the solute–solvent interaction, as well as potential challenges and pitfalls.

## Theory

Rather than present the fundamentals of persistent homology (see references [34–37] for introductions to the field), we will instead present a ‘greatest hits', where we will aim to give the reader a basic understanding, while paying little attention to the man behind the curtain.

### Persistent homology

**Fig. 1:**
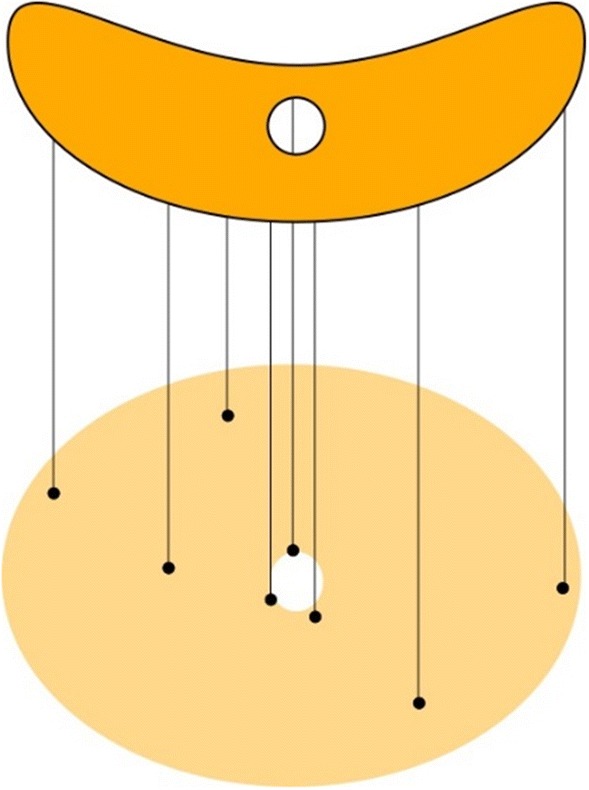
The sampling process. An illustration of the sampling process. A high-dimensional space (top) is sampled to the low-dimensional space (bottom)

In mathematics, homology is the general method of counting ‘holes’ in a space. Persistent homology is an extension developed to understand the holes in a data set. Consider the sampling process, as is illustrated in Fig. 1 individual observations are taken from some arbitrary space, and observed in some low-dimensional projection. We seek to understand the structure of the original space from its sampled points. However, the sampled points themselves have a topology which is trivial, there are $n_{points}$ connected components, and nothing else. To see a more interesting topology, we must ‘join up’ the points in some way. There are obviously a wide range of potential methods for this. In this work, we use the Vietoris–Rips (VR) complex. The VR complex requires a single parameter *δ*, and is defined on a data set $S$ as follows:

#### **Definition 1**

For every pair of points in (*x*, *y*) in *S*, if *d*(*x*, *y*)<, we draw a line between *x* and *y*. If every pair in a triplet (quartet, etc.) is connected, we draw the triangle (tetrahedron, etc.) between them.

**Fig. 2:**
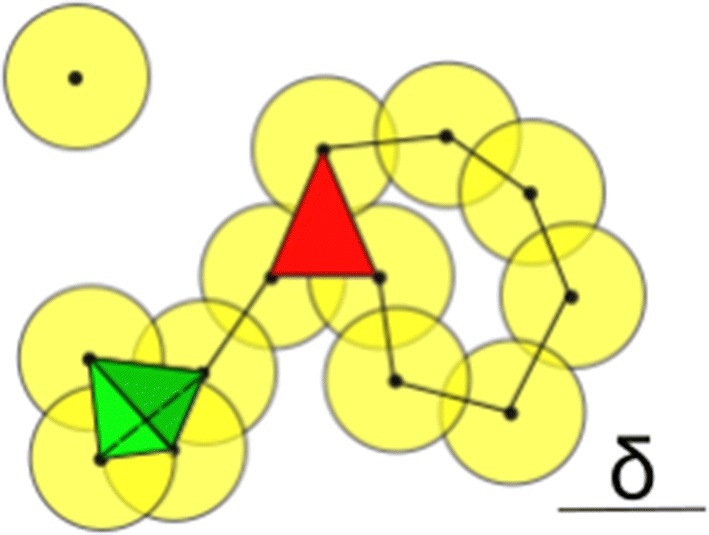
An example Vietoris*–*Rips complex. The Vietoris*–*Rips complex for the black dots, with parameter δ. Shaded circles are used to represent the distance used in the construction of the complex, and do not actually appear in the complex

An example data set and its associated VR complex can be seen in Fig. 2. The VR complex is relatively easy to compute, as it requires knowledge only about pairs of points. Given a VR complex, we can study its topology. As mentioned, homology is the method of ‘counting holes’ in a space. Mathematically, we calculate the *Betti numbers* $\beta_{n}$ of the space. For a given *n* a description of $\beta_{n}$ as well as the values for a sphere and torus, can be found in Table 1.

**Table 1:** Description of different Betti numbers $\beta_{n}$ and their associated values for a sphere and torus

$\beta_{n}$	Description	Sphere	Torus
0	Connected components	1	1
1	(Non-contractible) loops	0	2
2	Voids	1	1

The final ingredient of persistent homology is the ‘persistence’. One may ask—*What is the best value of to define a VR complex on a data set?* Persistent homology answers: *all of them*. By considering how the topology of the VR complex changes as we go through a range of *δ,* we hope to gain understanding as the structure of the underlying set of points. Any hole born at $t$ *must* be filled in by some $t '$. Therefore, we represent the persistent homology of a set of points by considering when topological features are born and when they die, in a *persistence diagram*.

**Fig. 3:**
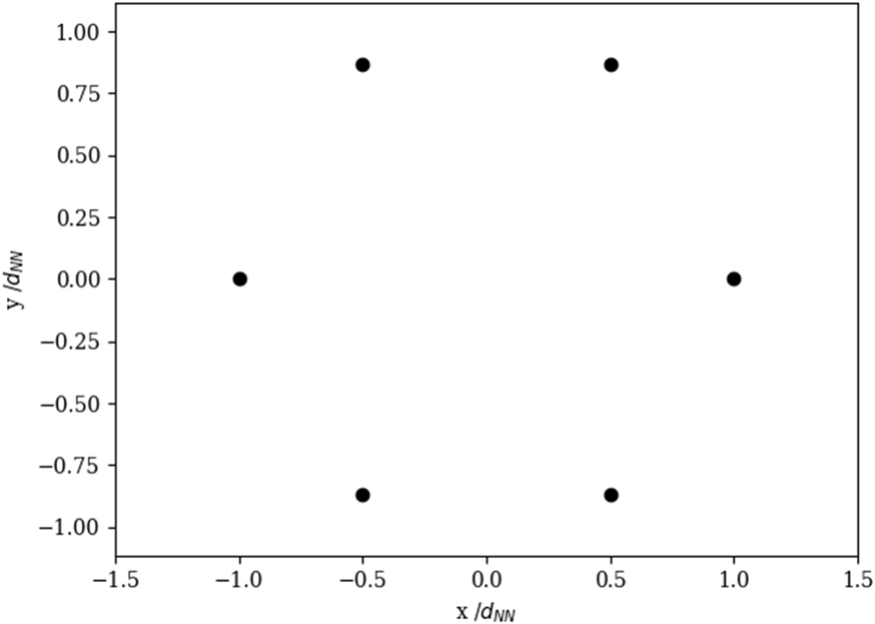
A hexagon

**Fig. 4:**
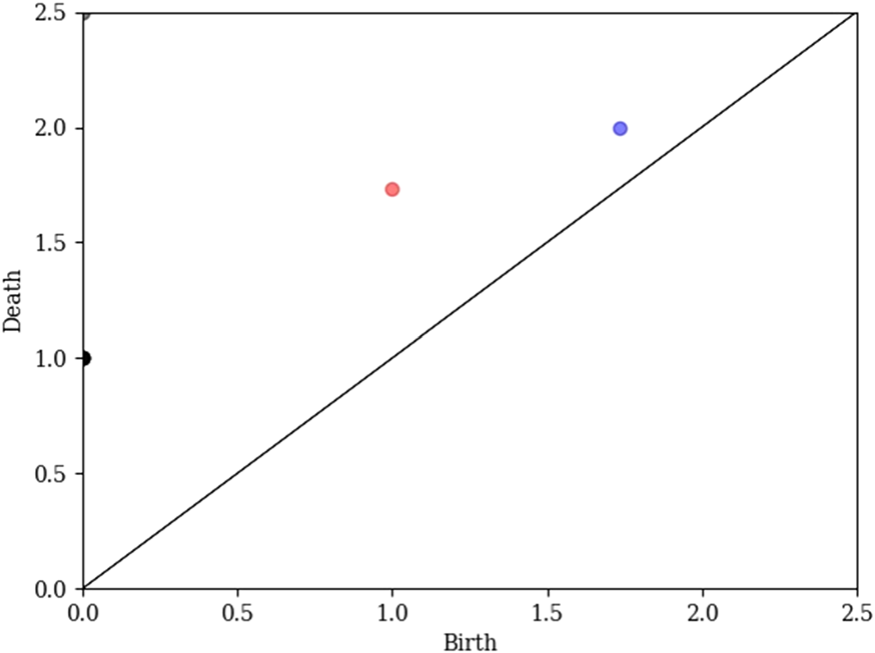
Persistence diagram of the regular hexagon. Black, red, and blue points represent zeroth, first and second degree homology respectively

For a regular hexagon, with nearest neighbour distance of *d* (Fig. 3), the persistence diagram can be seen in Fig. 4. At $\delta = 0$, we have 6 separate connected components. However, when $\delta = d$ these components merge to form a single component—5 (zeroth degree) components born at 0 die at *d*. Furthermore, in this merging, a loop (first degree component) is born. This loop persists until $\delta = \sqrt 3 d$ where now next-nearest neighbours join. This loop now dies, and the VR complex has the topology of a sphere. The sphere lives until $\delta = 2d$, where next-next-nearest neighbours join, filling in the sphere. An animation, demonstrating the various stages of the persistence diagram, can be found at [38]. All persistent homology calculations in this work were performed using the Gudhi library in Python [39].

### Persistence images

For this work, we will be trying to understand simulated water networks through the lens of persistent homology. Rather than comparing descriptors computed from single frames of simulation, which would be susceptible to noise, we would like to use a notion of *average* persistence. However, the persistence diagram is not well-suited to such a task (for more details we direct the reader to [40], particularly Fig. 3 therein). Therefore, there have been many attempts to construct vector representations of persistence diagrams that can have statistical techniques applied to them, including persistence landscapes [40, 41], kernel embeddings [42], and persistence images [32]. In this work, we use the persistence image, which transforms a single persistence diagram into a literal grayscale image. Furthermore, calculating the average of a set of images is as simple as finding the average value for each pixel. Lastly, persistence images are relatively simple to interpret, as they look similar to the persistence diagram.

The procedure of transforming a persistence diagram to a persistence image is as follows:

1. Select a single degree of homology
2. Transform each point of this degree from $\left( {b,d} \right)$ to $\left( {b,p} \right),$ where $p = d - b$
3. For each point $\left( {b,p} \right),$ define the function:
  $$g\left( {x,y} \right) = \frac{1}{{2\pi\sigma^{2} }}\exp \left( {\frac{{ - \left( {\left( {x - b} \right)^{2} + \left( {y - p} \right)^{2} } \right)}}{{2\pi\sigma^{2} }}} \right)$$
4. Multiply each function $g\left( {x,y} \right)$ by $\phi \left( {x,y} \right)$, where $\phi\left( {x,0} \right) = 0.$ This is done for stability reasons, and is discussed in more detail in [32]
5. Integrate $g\left( {x,y} \right)\phi\left( {x,y} \right)$, over a collection of pixels
6. The persistence image I is this discretisation of $g\left( {x,y} \right)\phi\left( {x,y} \right)$

**Fig. 5:**
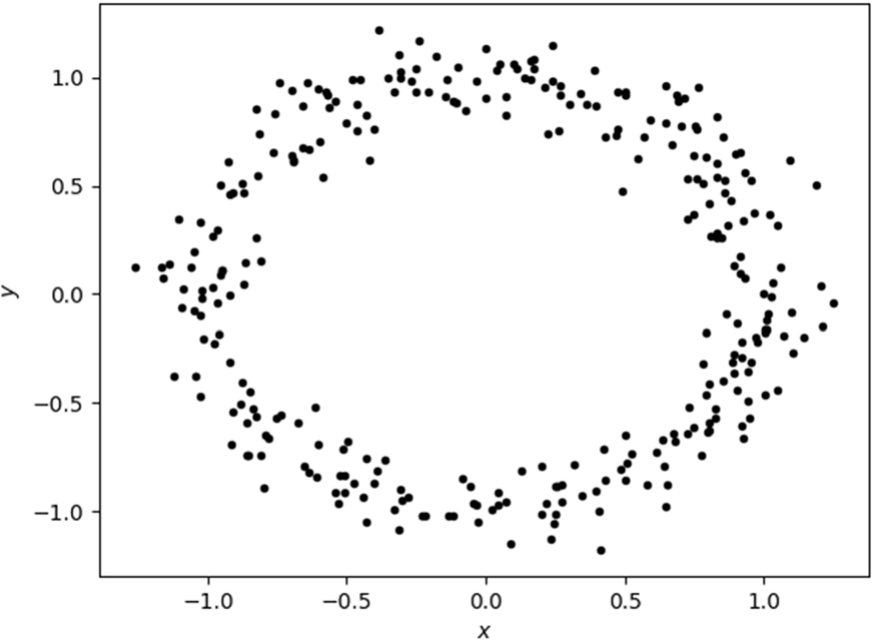
A noisy circular data set

**Fig. 6:**
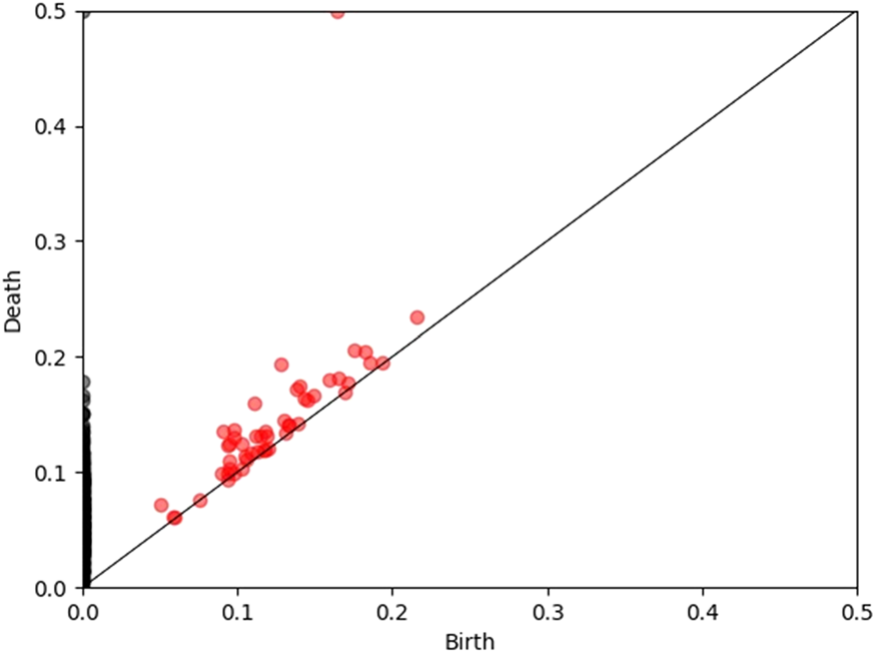
Noisy circle persistence diagram. The 2nd degree features have not been calculated for this example

**Fig. 7:**
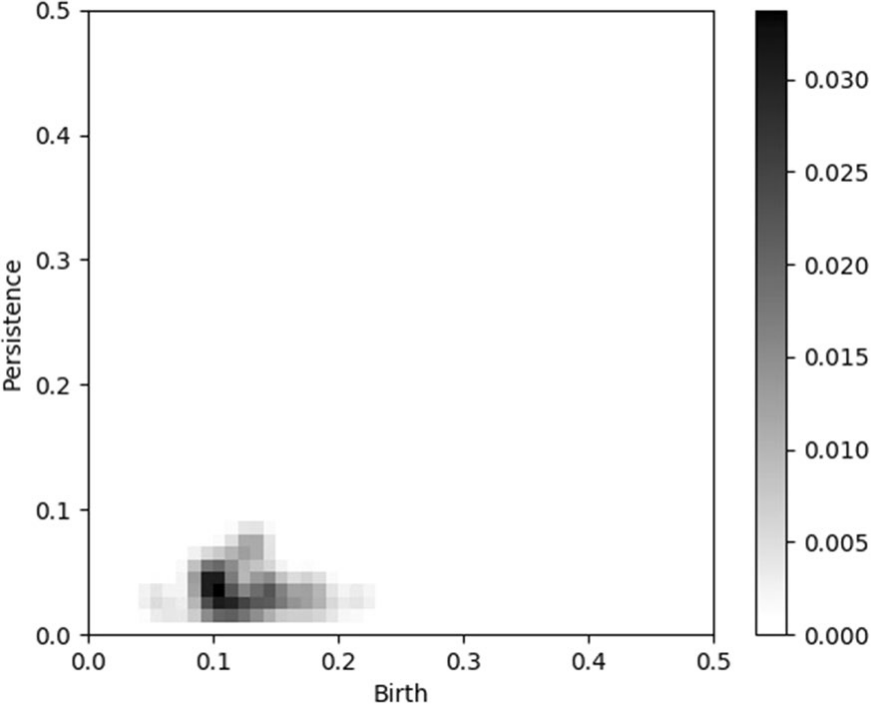
(First degree) persistence image for the noisy circle. The expected long-lived feature is present, but not visible as this corresponds to only one feature, as opposed to the thousands present at the maximum intensity

For a noisy circular data set (Fig. 5) the persistence diagram and 1st degree persistence image can be seen in Figs. 6 and 7 respectively. The persistence images in this work were computed using an in-house code, but can be calculated using libraries such as persim in Python. All persistence images in this code were calculated on a 50 × 50 grid of pixels.

## Simulation details

A brief summary is given here for the molecular dynamics simulations and for more information about simulation details, please refer to the Additional file 1.

### Atomistic potentials

The potentials used in this study are the commonly used TIP3P [43] and TIP4P/Ew [44] potentials, the SPC/E potential [45], and the more recent OPC potential. All of these potentials are fixed, therefore their dynamics are determined entirely by their intermolecular forces. Of these potentials, TIP3P and SPC/E are 3-site potentials, whereas TIP4P/Ew and OPC are both 4-site potentials. Table 2 contains details as to the parameters used for all the potentials. All simulations were performed using the AMBER 16 package [46]. Simulations were performed at a wide range of temperatures, at 1 atm pressure. This work only analyses the simulations performed at 300 K.

**Table 2:** The parameters of the various water models used in this study, and their physical meaning

Model	$q/e$	*l*/Å	*z*/Å	$\theta_{LJ} /$ °	*σ*_*LJ*_/Å	$\epsilon_{LJ}/{\text{kJmol}}^{- 1}$	$n_{atom}$
TIP3P	0.4170	0.9572	N/A	104.52	3.15061	0.636	4287
TIP4P/Ew	0.5242	0.9572	0.1250	104.52	3.16345	0.681	4254
SPC/E	0.4238	1.0000	N/A	109.47	3.16600	0.890	4287
OPC	0.6971	0.8724	0.1594	103.60	3.16655	0.89036	4302
SW	–	–	–	–	–	–	512

$\theta_{LJ}$ and $\epsilon_{LJ}$ are Lennard-Jones parameters for non-bonded interactions. The parameters for the SW potential are found in the main text

### The Stillinger–Weber potential

In contrast, the SW potential is a coarse-grained potential. Originally parameterised for Silicon in 1983 [33] the SW potential has been shown to be incredibly versatile, as can be seen from its general functional form [47]:

$$U = \mathop \sum \limits_{i,j}^{{}} U_{2} \left( {\varvec{r}_{ij} } \right) + \lambda \mathop \sum \limits_{i,j,k}^{{}} U_{3} \left( {\varvec{r}_{ij} ,\varvec{r}_{jk} } \right)$$

It is clear that the *λ* parameter allows the tuning of the relative strength of the 3-body interaction. The 2-body interaction models a steep repulsion at short distances, as well as a potential well:

$$U_{2} \left(r \right) = A\epsilon\left[{B\left({\frac{\sigma}{r}} \right)^{p} - \left({\frac{\sigma}{r}} \right)^{q}} \right]\exp \left({\frac{\sigma}{r - a\sigma}} \right)$$

Whereas the 3-body interaction can be considered to be an intermolecular bond stretch, as a harmonic spring as well as a distance factor:

$$U_{3} \left({r_{ij},r_{ik}} \right) = \epsilon [\cos_{ijk} - \cos_{0}]^{2} \times \exp \left({\frac{\sigma}{{r_{ij} - a\sigma}}} \right) \times \exp \left({\frac{\sigma}{{r_{ik} - a\sigma}}} \right)$$

In this work, we use the parameters *A *= 7.049556277, *B * = 0.6022245584, *p *= 4, *q *= 0, $\cos \theta_{0} = \frac{1}{3}$, *γ *= 1.2, and *a *= 1.8. All simulations of the SW potential were performed at the ambient temperature and pressure corresponding to the melting temperature at that particular *λ*.

## Persistent homology procedure for water simulations

### Persistent homology

Given a single frame of a simulation, we use the locations of the oxygen atoms as our point cloud. This leads to a substantially quicker computation time, as we are reducing the number of points in our system by 2/3. This decision also makes sense from a theoretical perspective, namely that it is the tetrahedral nature of the oxygen lattice which is of interest, and including the hydrogen atoms as equal in the persistent homology would likely ‘wash out' this information, and instead simply capture the persistent of densely sampled Euclidean 3-space. We note that this procedure is much simpler than the element specific persistent homology of Cang and Wei [16] and the multiparameter persistence of PHoS developed by Keller, Lesnick and Willke [48]. However, the relative simplicity of our systems compared to the drug-like biomolecules used in their work allows us to use such a simple procedure. Furthermore, our procedure naturally extends to the coarse-grained SW potential. For each degree of homology separately, we calculate the persistent homology for every frame of simulation, before converting each persistence diagram into a persistence image.

### L1-normalised persistence images

One of the fundamental properties of a potential descriptor for water structure is that it is size-independent. Provided two systems are large enough such that bulk behaviour dominates, we would like to be *unable* to separate two systems of different sizes using the structural descriptor. This is a problem for persistent homology, where the number of persistent features is *clearly* dependent on the number of points in the system. In persistence images, this property is the integral of the image. We therefore define the L_1_-normalised persistence image (L1NPI) as:

$${\text{L}}1{\text{NPI}}\left[ {i,j} \right] = \frac{{I\left[ {i,j} \right]}}{{\mathop \sum \nolimits_{i,j} I\left[ {i.j} \right]}}$$

where $I\left[ {i,j} \right]$ is the value of the persistence image at the pixel with index $\left[ {i,j} \right].$ The most significant consequence of this definition is that the mean LINPI is not equal to the L_1_-normalised mean persistence image. We do not explore this discrepancy in our work.

### Comparison to other techniques

The radial distribution function (RDF) is a standard tool when analysing simulations of materials, such as the water networks discussed in this work. The RDF describes the relative density of water molecules as a function of distance, and allows the discovery of solvation shells. The RDF has previously been used to compare different water models such as in [3], where it was shown that the slight differences in Lennard-Jones and Coulombic terms led to pronounced changes in density of second-nearest neighbours. An extension of the RDF, the spatial distribution function (SDF) was developed, which does not integrate out the angular distribution in the manner of the RDF. The SDF, when applied to SPC/E water, led to the discussion of two different motifs, a temperature independent tetrahedral water, and a non-tetrahedral structure that appeared to vary with temperature [4].

Persistent homology is a more complex and rich tool for analysing these structures. Rather than simply studying the relative positions of pairs of water molecules, the simplicial complex required in persistent homology contains information about groups of water molecules. For example, the presence of the triangle *abc* in the simplicial complex requires all pairs *ab*, *ac*, *bc*, to be within a particular distance of each other. This leads to information that can be related to the RDF—the nearest neighbour distance can be estimated using zeroth-degree homology—but also information that is not so easily extracted from either the RDF or SDF—such as the presence of rings of water structures.

## Results and discussion

### Comparison of persistence images and L1NPIs

**Fig. 8:**
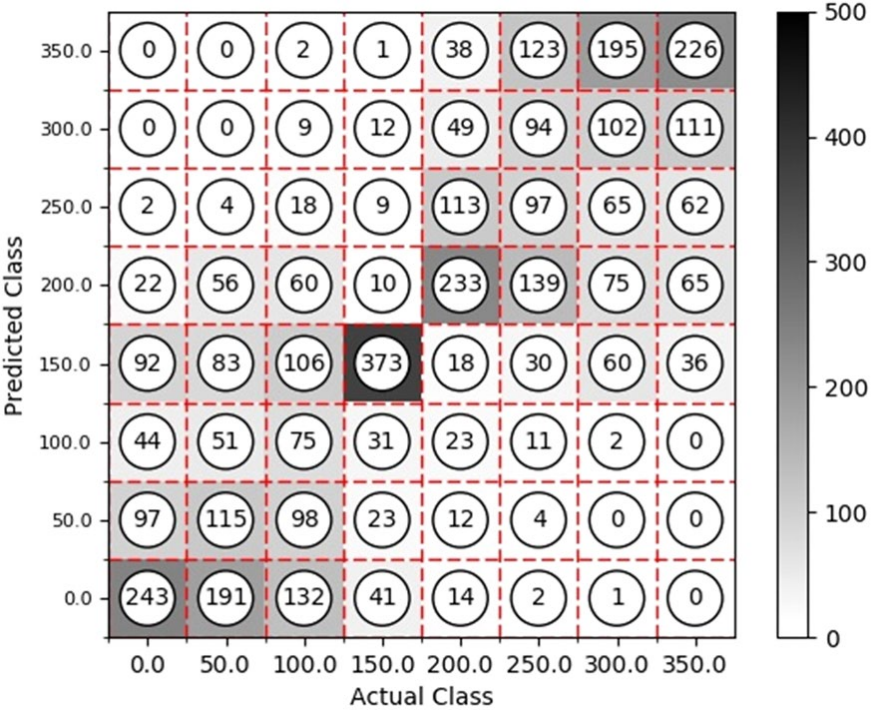
Confusion matrix for linear SVM on persistence images. Classes are defined as the number of water molecules removed from the system, with class 0 containing 4287 water molecules

**Fig. 9:**
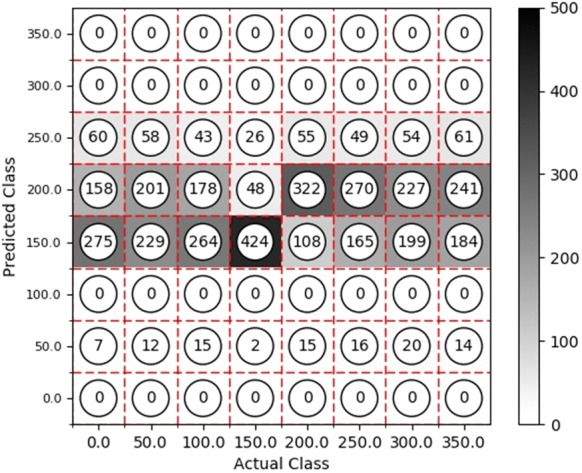
Confusion matrix for linear SVM classifier on L1NPIs. Classes defined analogously to Fig. 8

To demonstrate the usefulness of the L1NPI versus the standard persistence image, we investigate the performance of a linear SVM classifier on systems of the same potential, with different numbers of water molecules. Firstly, the L1NPI matrix is flattened into a high-dimensional vector in ${\mathbb{R}}^{2500}$. A size-independent descriptor would perform badly on this classification task, as it would not be able to distinguish between the only difference in the systems. We present the confusion matrices for these classifiers for both the first degree persistence images and L1NPIs for simulations of TIP3P water at 300K with varying numbers of water atoms in the system in Figs. 8 and 9 respectively. It is clear that the standard persistence image SVM performs well in this classification task, and that it can distinguish between systems based on the number of water molecules present. However, the L1NPI performs much worse at this task, and can be seen to be essentially randomly guessing between 3 classes.

**Fig. 10:**
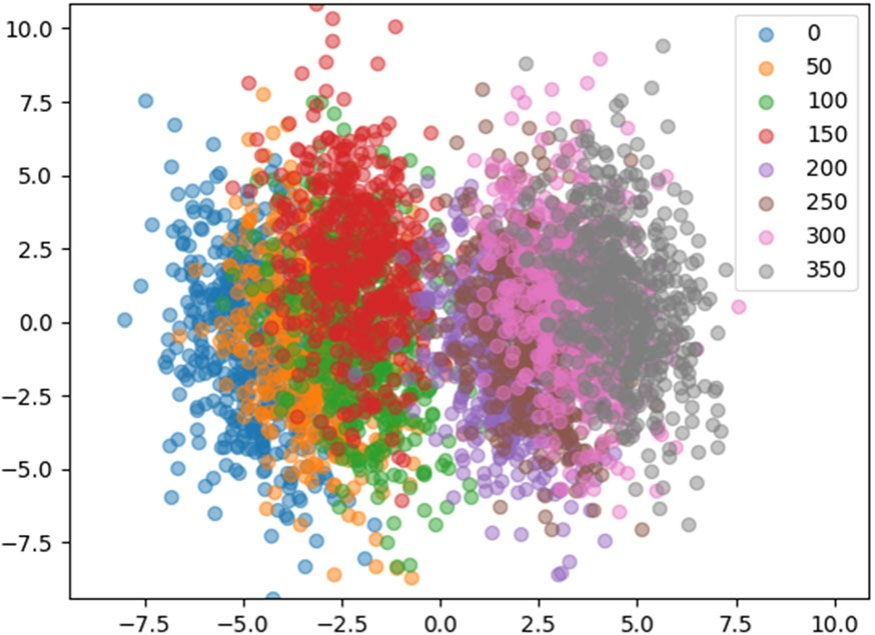
PCA of first degree persistence image space for different numbers of water molecules in TIP3P. Classes are defined by the number of waters removed from the system, where 0 refers to a system with 4287 water molecules

**Fig. 11:**
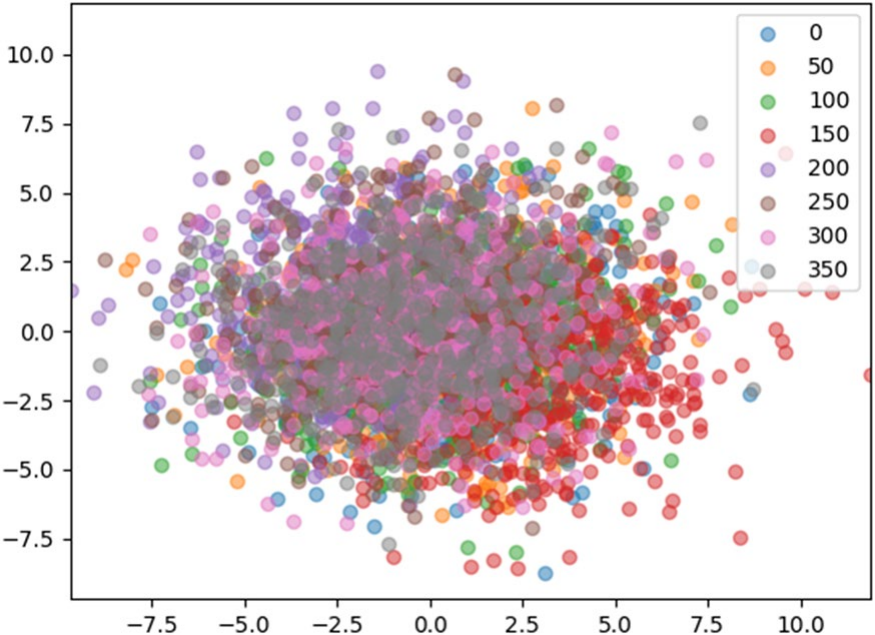
PCA of first degree L1NPI space for different numbers of water molecules in TIP3P. Classes defined analogously to Fig. 10—PCA of first degree persistence image space for different numbers of water molecules in TIP3P. Classes are defined by the number of waters removed from the system, where 0 refers to a system with 4287 water molecules

To explain the performance of these classifiers, we have performed PCA on the persistence image and L1NPI spaces, with the two-dimensional projections in Figs. 10 and 11 respectively. Clearly, the persistence images form a trend with the number of water molecules in the system, which is far less prominent in the L1NPI. We can conclude from this that the L1NPI is much more size independent than the standard persistence image. However, this is not a total size-independence, as there is likely to always be some finite-size effect in persistent homology.

### Comparison of atomistic potentials

**Fig. 12:**
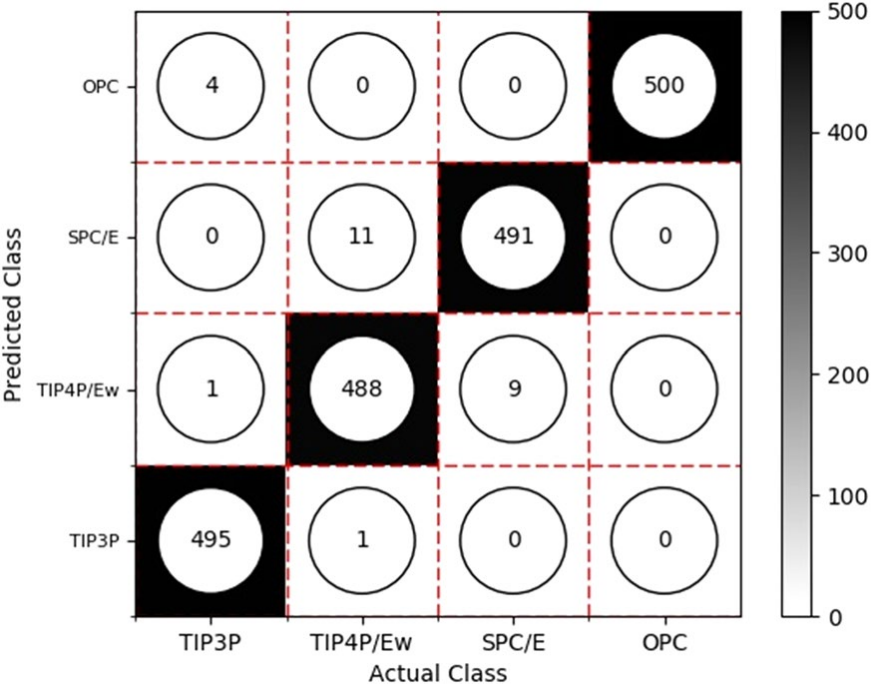
First degree L1NPI confusion matrix. The 1st degree L1NPI confusion matrix for a linear SVM classifier on different atomistic models

**Fig. 13:**
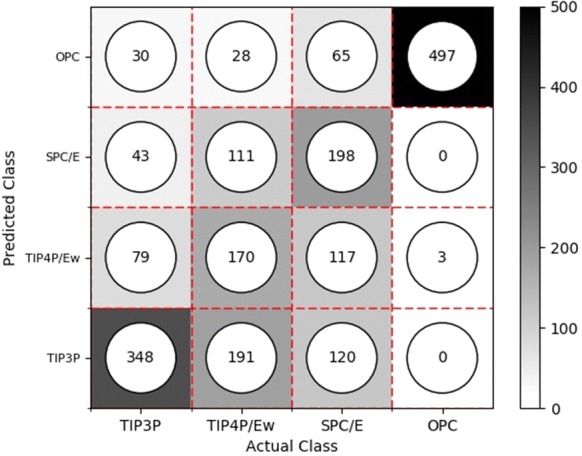
Second degree L1NPI confusion matrix. The 2nd degree L1NPI confusion matrix for a linear SVM classifier on different atomistic models

The SVM classifier formalism can also be used to analyse differences between the atomistic potentials. We would expect any differences to be subtle here, as they are ostensibly modelling the same system. The confusion matrices for first and second degree homology at 300 K for these systems can be seen in Figs. 12 and 13 respectively. First degree homology is able to correctly classify different atomistic potentials with 98.7% accuracy. In contrast, second degree homology performs with 60.6% accuracy. Interestingly, this accuracy is not consistent amongst the potentials studied. In particular, OPC is correctly classified 99.4% of the time, whereas TIP4P-Ew has only 34% accuracy.

**Fig. 14:**
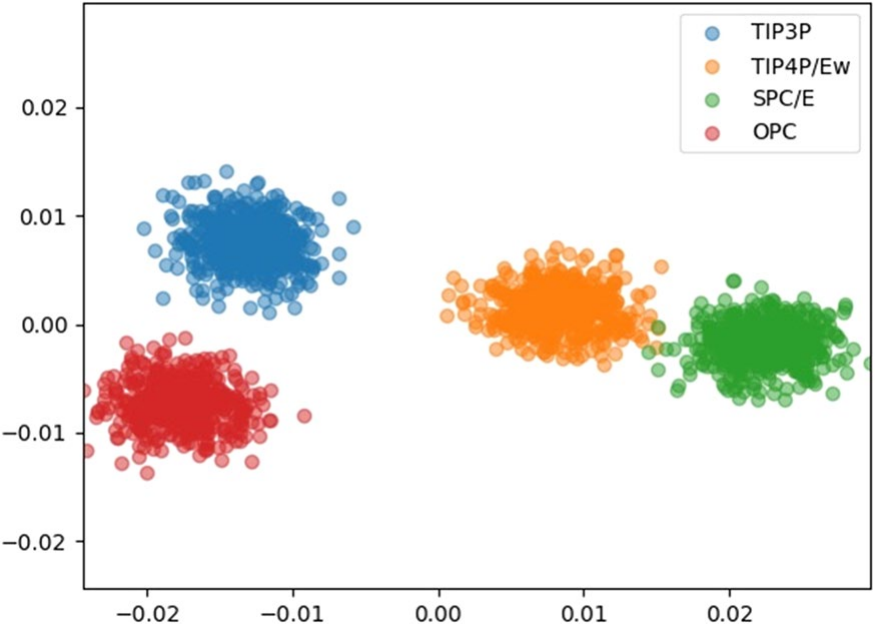
First degree L1NPI principal components. Projection onto the first two principal components for first degree L1NPI space

**Fig. 15:**
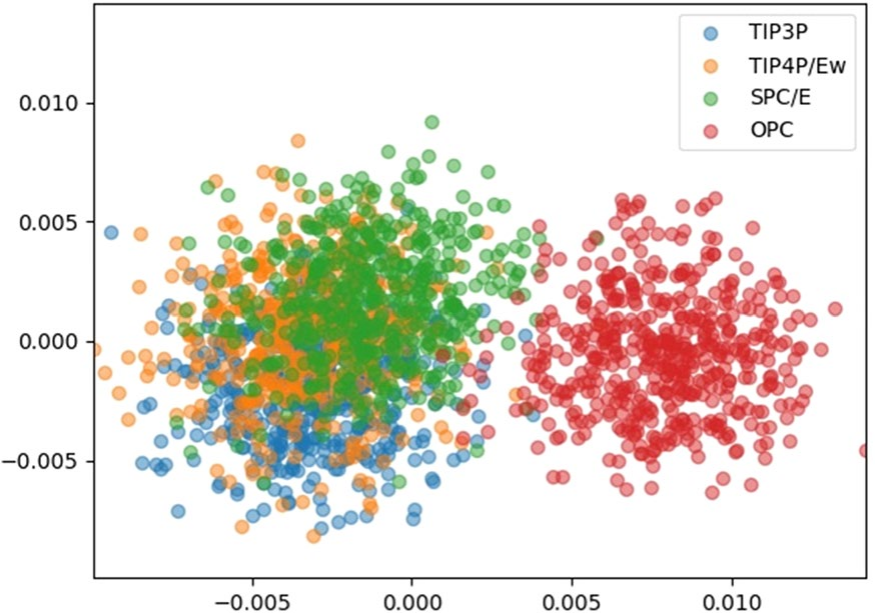
Second degree L1NPI principal components. Projection onto the first two principal components for second degree L1NPI space

We are able to analyse these systems further using principal component analysis (PCA). We project the L1NPI vectors onto the first two principal components of the system, which can be seen in Figs. 14 and 15 for first and second degree L1NPIs respectively. Firstly, we can state that the major differences in structure are clearly coming from first degree homology. This is reflected in the greater separations of potentials in first degree homology. Furthermore, we recover the well-known fact that TIP4P/Ew and SPC/E are more similar than TIP3P.

**Fig. 16:**
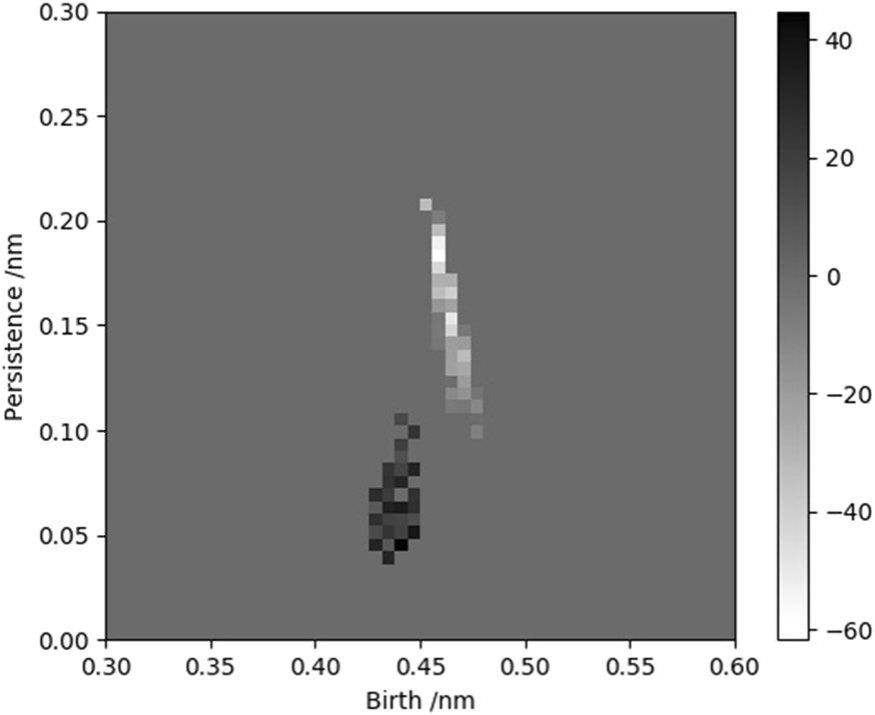
Separating hyperplane for OPC. The separating hyperplane for distinguishing OPC from other atomistic models, in second degree homology

Using PCA, it is immediately apparent that OPC can be distinguished in second degree homology, whereas the other potentials studied cannot. Using the discovered coefficients of the SVM classifier, we are able to recover the L1NPI-like image that represents the separating hyperplane (such an image is L1NPI-like as pixels are allowed to take negative values, unlike in a L1NPI. It is the absolute value of the pixels in the separating hyperplane image that corresponds to their importance to the classifier). For OPC, this hyperplane image can be seen in Fig. 16. It is clear that the distinguishing characteristics for OPC is the presence of more points of low persistence, with a lack of points of high persistence.

### Comparison of series of Stillinger–Weber potentials

**Fig. 17:**
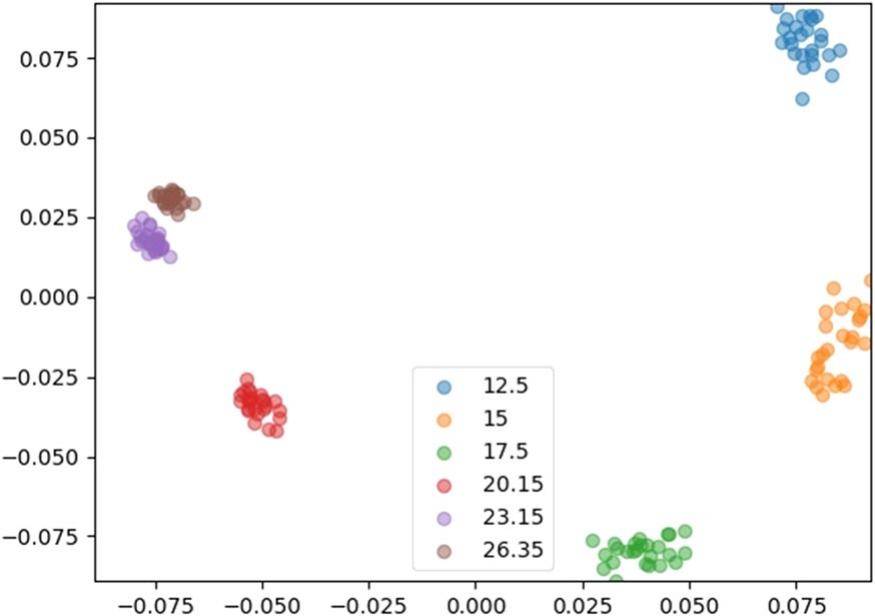
Principal components for SW potential first degree L1NPI space. The projection of the first degree L1NPI space onto its first two principal components, for a range of *λ* in the SW potential

**Fig. 18:**
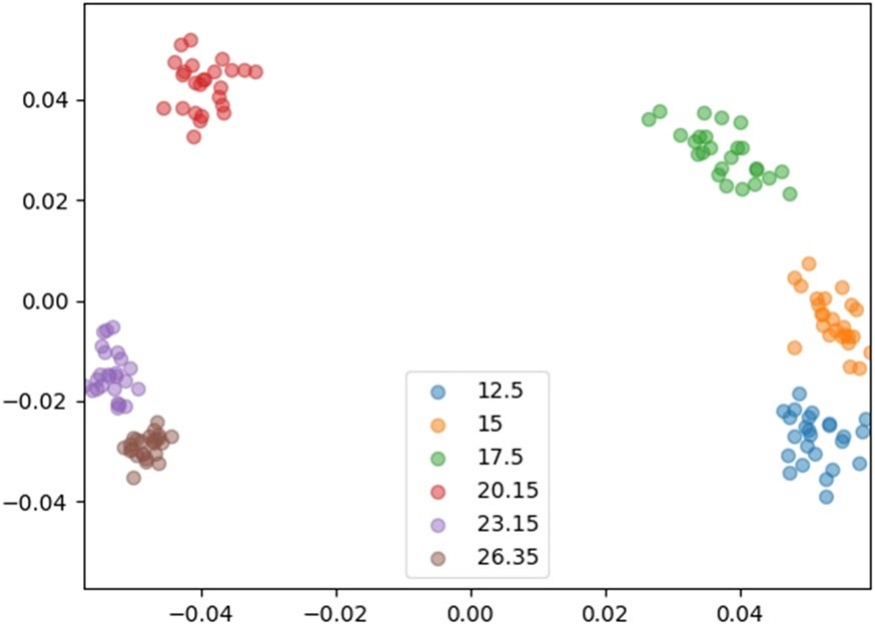
Principal components for SW potential second degree L1NPI space. The projection of the second degree L1NPI space onto its first two principal components, for a range *λ* of in the SW potential

As the SW simulations derive from a series of related potentials differing in one main parameter we can investigate how the topological analysis is related to this parameter. A series of simulations for the Stillinger–Weber potential were performed at different values of the *λ* parameter. The projection of L1NPI space onto its first two principal components can be seen in Figs. 17 and 18 for first and second degree homology respectively. Firstly, we note that the differences in L1NPI space are much more pronounced with the value of *λ* changing than previously shown for the different atomistic water potentials. This is expected, as the different atomistic potentials are ostensibly modelling the same system, whereas the SW potential has been derived to simulate vastly different systems depending on *λ*. We also see the same behaviour that differences are more pronounced in first than second degree homology. This can be explained by the following argument. In a relatively dense point cloud, such as the ones being studied in this work, it is a reasonable first approximation to associate the nearest-neighbour distance to the birth value of first degree homology, next-nearest neighbour distance to the death value of first degree homology, and the birth value of second degree homology, and so on. It is clear that as we look at interactions beyond nearest neighbour, interactions become less directional, and the distribution of distances becomes wider [49]. This is reflected in an increase of similarity of persistence.

It is also interesting to note that the distribution of points in L1NPI space narrows as *λ* increases. This suggests a reduction in the variance of the persistent homology. As *λ* increases, the relative strength of the three-body interaction defined in the SW potential increases. This leads to a reduction in the variance of next-nearest neighbour distances, which is then reflected in the persistence.

### Comparison of atomistic and Stillinger–Weber potentials

**Fig. 19:**
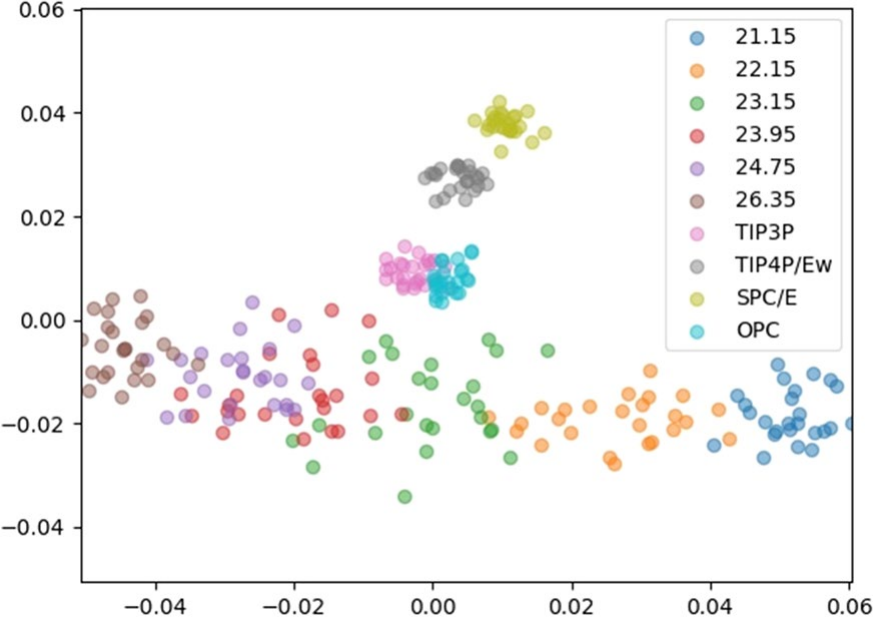
Principal components for first degree L1NPI space. The projection of the first degree L1NPI space onto its first two principal components for both a range of *λ* in the SW potential and a range of atomistic potentials

**Fig. 20:**
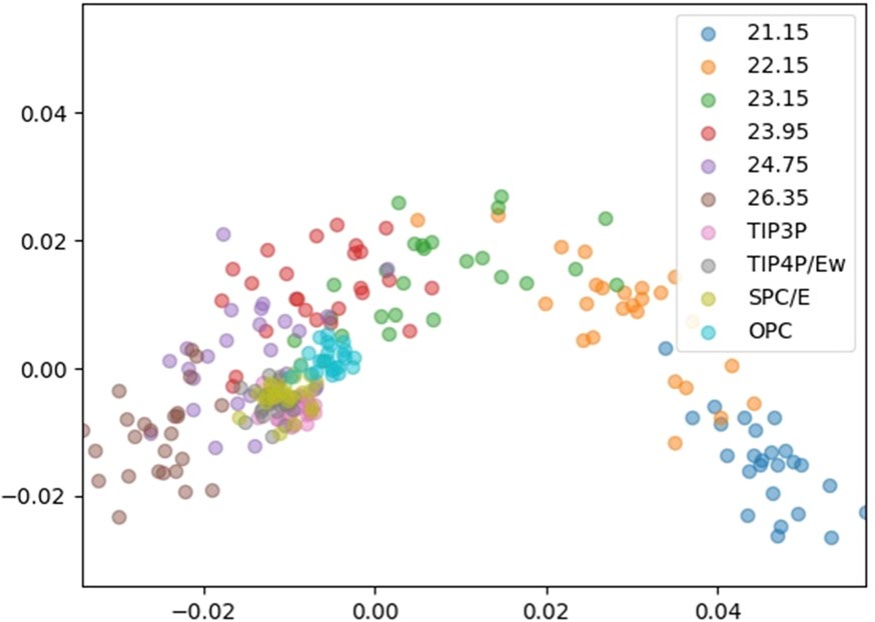
Principal components for second degree L1NPI space. The projection of the second degree L1NPI space onto its first two principal components for both a range of *λ* in the SW potential and a range of atomistic potentials

Lastly, we will present a comparison between the atomistic and SW potentials using the L1NPI formalism. Figures 19 and 20 show the first two principal components of first and second degree L1NPI space for a selection of values of *λ* and the previously used atomistic potentials. In both degrees, it is clear that the atomistic potentials do not lie on the same line as the SW potential simulations, and as mentioned before they are closer to each other than variations in *λ*. Again, this suggests that the differences in atomistic potential lead to more subtle changes in structure than altering *λ*, and the differences in structure between atomistic potentials are not the same as the differences in structure in SW potential simulations.

We note that in first degree homology, the atomistic potential simulations are closest to the value of *λ* = 23.15. This is the value of *λ* that is considered to lead to water-like structures, as it reproduces the density profile of water on a range of temperatures. Interestingly, this is not the case in second degree homology, where the L1NPI descriptor suggests that the atomistic systems are more similar to *λ* = 23.95, with OPC being closest to *λ* = 23.15. This separation of degrees of homology is a useful property of the L1NPI analysis, where we are able to say that although the atomistic structures have the same ‘loops’ of the SW structures, they do not match the ‘holes’, with OPC being the closest.

We finally return to the size-independent nature of our L1NPI descriptor. Whereas the atomistic potentials have in excess of 4000 water molecules, the simulations of the SW potential have 512. However, the L1NPI descriptor can be used to compare such systems, irrespective of system size. We do note that there is one consequence of size in the L1NPI formalism, which can be seen in the PCA images. Namely, systems with more molecules lead to a tighter distribution of points in L1NPI space. Considering a single frame of a simulation, we note that more molecules will lead to more points in the persistence diagram, which will become more ‘filled in’. This implies that the individual persistence diagrams (and therefore images and L1NPIs) will be more similar to each other in simulations with a larger number of particles. Therefore, the L1NPI descriptor is not entirely size-independent, although it is far more size independent than other persistence representations.

## Conclusion

We have derived a new descriptor for water network structure, using topological data analysis. By applying persistent homology, the study of holes in data, to the point cloud defined by oxygen atom coordinates, we are able to gain insight as to what distinguishes various structures created by different intermolecular potentials. Whereas more commonly used techniques, such as persistence landscapes [40] are unable to be used on systems of widely varying sizes, we have shown that our technique, the L_1_-normalised persistence image (L1NPI) is relatively size-independent.

We first applied the L1NPI formalism to four commonly used atomistic potentials: TIP3P, TIP4P/Ew, SPC/E and OPC. We were able to determine that first degree homology (i.e. loops) were enough to distinguish between these potentials, even with a relatively simple linear support vector machine. In contrast, second degree homology (holes) was only able to distinguish between OPC and the other models. We consider this to be a consequence OPC’s rather unique parameterisation technique. We are also able to show that TIP4P/Ew and SPC/E are more similar than the other atomistic models, purely based on their proximity in L1NPI space.

We then investigated a series of Stillinger–Weber potentials. By tuning the parameter *λ*, the relative strength of the three-body interaction can be altered. The L1NPI formalism showed that differences in structure caused by changing *λ* are much more pronounced than those found in the atomistic potentials. Furthermore, we were able to relate properties such as nearest neighbour distances to observations in L1NPI space.

We finally compared the atomistic systems to the Stillinger–Weber potential series. We noticed that in first degree homology, the atomistic structures are closest to the widely accepted value of *λ* = 23.15. In contrast, second degree homology suggests that the structures are closer to slightly higher values of *λ*, with OPC being closest to 23.15. Furthermore, by comparing systems of widely different sizes (512 vs. 4000 water molecules), we show that the L1NPI formalism is size-independent.

It would be interesting to study generalisations of the persistence image to other means, rather than simply the L1 norm, as a method of future work. The use of generalised mean-based descriptors is well established, such as in [50, 51], and we feel that different means could be able to account for other discrepancies than system size.

We conclude by discussing the application of the L1NPI formalism to the solubility problem, Although it is widely accepted that there is a need to produce better models (as evidenced by the ‘Solubility Challenge’ [52, 53]) models are still unable to accurately predict water solubility [53]. We feel that a large amount of research is invested in producing models with more complex designs. This, coupled with the lack of high-quality solubility data, leads to overfitted models, as well as poor interpretability. However, the L1NPI formalism could be applied to solute–solvent systems, and in particular differences in L1NPIs could be related to perturbations of the water network. We plan to expand upon this problem further in a future publication.

## Additional file

**Additional file 1.** Python programs for computation of persistence.

## Data Availability

All software used in this work is either open-sourced, or a reference given to an open-source implementation. Key software components are included in the supplementary material. Copies of coordinate files for the simulations are available on request.
