# Correction to: A new topological descriptor for water network structure

**DOI:** 10.1186/s13321-019-0377-0

**Published:** 2019-08-07

**Authors:** Lee Steinberg, John Russo, Jeremy Frey

**Affiliations:** 1 School of Chemistry, University of Southampton, Southampton, SO17 1BJ UK; 2 School of Mathematics, University of Bristol, Bristol, UK

## Correction to: J Cheminform (2019) 11:48 10.1186/s13321-019-0369-0

It was highlighted that the original article [1] was missing Additional files 2 and 3.

This Correction article includes the missing Additional files 2 and 3:

- Supporting Information.docx
  - Details as to simulations
- Utilities.zip
  - Python scripts for running analysis

## Additional files

**Additional file 2.** Simulations details.

**Additional file 3.** Python scripts for running analysis.

## References

[1] Steinberg L, Russo J, Frey J (2019). A new topological descriptor for water network structure. J Cheminform.

