# Supplementary material for: A new topological descriptor for water network structure
Source: J Cheminform. 2019 Jul 10;11:48. doi: 10.1186/s13321-019-0369-0 (PMC6617667; doi:10.1186/s13321-019-0369-0)
Supplement: Supplementary file 1 — Additional file 1. Python programs for computation of persistence. [file 13321_2019_369_MOESM1_ESM.docx]

**A new topological descriptor for water network structure: supporting information**

**Simulation Details**

**Atomistic potentials**

| Potential | Number of Water Molecules* |
| --- | --- |
| TIP3P | 4287 |
| TIP4P/Ew | 4254 |
| SPC/E | 4287 |
| OPC | 4302 |

* This was the ‘control’ number of waters used for the experiment where water molecules were removed from the system.

Simulations were carried out at a range of temperatures. The simulations used in the work were all performed at 300K, 1atm pressure.

Systems were first created using the SOLVATEBOX utility provided with AMBER. Temperature was equilibrated over 100ps, with a time interval of 2fs. Pressure was then equilibrated over 500ps, with the same timestep. Then production runs were performed for 4ns in the NVT ensemble. Representative frames were taken every 2ps.

**Stillinger-Weber Potentials**

All SW potential simulations contained 512 water molecules.

First define the time taken for a water molecule to diffuse over 3Å as τ (this is determined from the diffusion coefficient, refer to [1] for more details. Simulations were equilibrated for at least 100τ, and configurations sampled every 50τ to ensure they were independent.

**References**

[1] Water-like anomolies as a function of tetrahedrality, J. Russo, K. Akahane, H. Tanaka, *PNAS*, 115 (15), 2018
